# Update on acquired hypogonadism in men living with HIV: pathogenesis, clinic, and treatment

**DOI:** 10.3389/fendo.2023.1201696

**Published:** 2023-06-26

**Authors:** Sara De Vincentis, Vincenzo Rochira

**Affiliations:** ^1^ Unit of Endocrinology, Department of Biomedical, Metabolic and Neural Sciences, University of Modena and Reggio Emilia, Modena, Italy; ^2^ Unit of Endocrinology, Department of Medical Specialties, Azienda Ospedaliero-Universitaria of Modena, Modena, Italy; ^3^ Clinical and Experimental Medicine PhD Program, University of Modena and Reggio Emilia, Modena, Italy

**Keywords:** frailty, sex steroids, testosterone, SHBG, functional hypogonadism

## Abstract

Hypogonadism is a frequent finding among men living with HIV (MLWH) and it seems to occur earlier in comparison with the general male population. Although the prevalence of hypogonadism in MLWH has significantly lowered thanks to advancements in medical management, it remains high if compared with age-matched HIV-uninfected men, ranging from 13% to 40% in the age group of 20-60 years. Signs and symptoms of low serum testosterone (T) in MLWH are cause of concern since they are non-specific, of mild-to-moderate degree, and often overlapping with those of infection *per se*. For these reasons, hypogonadism can be underestimated in the absence of targeted laboratory blood examinations. With regard to the etiological factors involved in the T decrease, emerging evidence has suggested the functional nature of hypogonadism in MLWH, pointing out the mutual relationship between sex steroids, health status, comorbidities, and HIV-related factors. In agreement with this hypothesis, a therapeutic approach aiming at improving or reversing concomitant diseases through lifestyle changes (e.g. physical activity) rather than pharmacological T treatment should be theoretically considered. However, considering both patient’s barriers to lifestyle changes to be maintained overtime and the lack of evidence-based data on the efficacy of lifestyle changes in normalizing serum T in MLWH, T therapy remains an option when other non-pharmacological interventions are ineffective as well as for all other functional forms of hypogonadism. From this perspective, the traditional therapeutic management of male hypogonadism in MLWH, especially the role of T supplementation, should be revised in the light of the probable functional nature of hypogonadism by considering a good balance between benefits and harmful. This narrative review presents an overview of current knowledge on hypogonadism in MLWH, deepening the factors driving and taking part in T decrease, providing advice for the clinical approach, and underlining the importance of individualized treatment aiming at optimizing non-gonadal comorbidities and thus avoiding over-, or even unnecessary, treatment with T.

## Introduction

A link between hypogonadism and HIV has been traced since the first case reports of infection. Over the last decades, the life expectancy of MLWH has improved thanks to the medical progresses (1, 2). First, the introduction of highly active antiretroviral therapy (HAART) in the early 90s has drastically impacted the natural history of HIV infection, moving from a rapidly fatal illness to a chronic disease (3). As a consequence, the clinical presentation of MLWH has profoundly changed over time and the historical HIV-related complications have been progressively overtaken by emerging chronic non-infectious comorbidities (4, 5). Hence, if before the advent of HAART the therapeutic goal for MLWH was patients’ survival, in the post-HAART era, the focus of both clinical and research approaches shifted to the care of chronic comorbidities and the progressive HIV population aging (6, 7). This multimorbidity is one of the main characteristics of ‘frailty’ that depicts the condition of poor health status recognized in MLWH, even young-to-middle aged (4, 5), as well as in the elderly HIV-uninfected population (4, 5, 7, 8). As MLWH live longer than in the past, their total number as well as the number of older adults living with HIV aged 50 years or older is constantly increasing worldwide (9), the latter reaching 8.1 million in 2020 (considering both sexes), according to UNLAIDS (10). Thus, the management of comorbidities is challenging in MLWH. Together with chronic liver, renal, cardiometabolic, and oncological comorbidities, the endocrine system is not spared from the long-term effects of both virus and antiretroviral drugs (1, 2, 5, 11, 12). Notwithstanding the changes and the general improvement obtained in the control of the disease, several endocrine diseases such as growth hormone deficiency and diabetes mellitus are considered more prevalent in HIV population than age-matched uninfected subjects (5, 13). Among these, an impairment of the hypothalamic–pituitary-gonadal axis is frequently reported in MLWH causing testosterone (T) deficiency that remains one of the most frequent endocrine complaints in this population (11, 14–16).

In the literature several studies have explored the prevalence of hypogonadism in MLWH reporting highly variable findings and some peculiar issues should be considered when comparing these studies with each other. Indeed, this heterogeneity can be explained by different diagnostic cut-offs adopted to define hypogonadism depending on total T and/or free T, different serum T assays, and different mean age of enrolled patients (16, 17). Overall, it seems that the prevalence of overt biochemical hypogonadism (i.e. low T concentrations) among MLWH has been decreasing in recent years thanks to improvement of their general health status (17), although the finding of subclinical hypogonadism (i.e. normal T concentrations combined with increased gonadotropins) remains quite common in these patients (18). The decreasing trend of the prevalence of T deficiency reflects the timeline of the medical advancements obtained in the control of HIV infection. In this view, the introduction of HAART represents a sort of watershed in the history of HIV medicine. Concerning the pre-HAART era, published studies on the prevalence of male hypogonadism reported more heterogeneous results in comparison to recent studies, further suffering of the same abovementioned methodological limitations. Overall, based on *a posteriori* estimate, the prevalence of hypogonadism in MLWH before HAART was about 30- 40% and it was associated with the progression of HIV infection to AIDS as well as to the concomitant development of AIDS related wasting syndrome (19–24). Prevalence data of the post-HAART period, on which this systematic review is focused, will be presented in detail in the specific paragraph.

From a clinical standpoint, the diagnosis of hypogonadism in MLWH poses challenges since signs and symptoms of low serum T are non-specific, of mild-to-moderate degree, and often overlapping with those of other diseases in MLWH. For these reasons, hypogonadism can be underestimated in the absence of targeted laboratory blood examinations (14, 16). As largely emphasized by guidelines on male hypogonadism (25, 26) as well as by recent reviews (16, 27), original studies (18, 28), and meta-analysis (17), a detailed and accurate evaluation of the functionality of the pituitary-gonadal axis in MLWH should require the measurement of the sex hormone-binding globulin (SHBG) together with serum total T and gonadotropins. Notwithstanding these recommendations, at present, however, only few research studies have explored gonadal function in MLWH with the combination of serum sex steroids, SHBG and gonadotropins. In the daily clinical practice, even more than in research settings, there could be further limitations in having access to complete and performant hormonal assays.

In male subjects circulating T declines with aging (29) and the same trend is observed also in MLWH but it seems to occur earlier in comparison to the general population (14, 16, 17, 30, 31). Even though the underlying pathogenetic mechanisms are still to be fully elucidated, available data suggests that this progressive serum T decline in MLWH has a multi-factorial etiology where both direct viral effects on testicular tissue and undesired effects of HAART may contribute to affect T levels (14, 16, 30, 32). Recently, new insights have been added in this topic proposing HIV-related hypogonadism as a form of functional hypogonadism secondary to poor health status and chronic comorbidities (31, 33). Hence, a poor health status seems to be associated to low T in older, frail men even outside the context of HIV infection (34–37). In particular, low T levels are associated to several systemic diseases (38) such as obesity (39), diabetes mellitus (40), dyslipidemia, and metabolic syndrome (41). These forms of male hypogonadism are considered as strictly related to poor health, secondary to other morbidities and potentially reversible, and endocrinologists tend to classify them as a sort of functional hypogonadism (38, 42, 43). Actually, there is emerging evidence confirming the role of serum T as marker of frailty and the mutual relationship among comorbidities, health status, and sex steroids even in MLWH (31, 33, 44). In agreement with this hypothesis, a therapeutic approach aiming at improving or reversing concomitant diseases through lifestyle changes (e.g. physical activity) rather than pharmacological T treatment should be theoretically considered. However, given both patient’s barriers to lifestyle changes to be maintained overtime and the lack of evidence-based data on the efficacy of lifestyle changes in normalizing serum T in MLWH, T therapy remains an option when other non-pharmacological interventions are ineffective as well as for all other functional forms of hypogonadism. From this perspective, the traditional therapeutic management of male hypogonadism in MLWH, especially the role of T supplementation, should be revised in the light of the probable functional nature of hypogonadism by considering a good balance between benefits and harmful. This narrative review presents an overview of current knowledge on hypogonadism in MLWH, providing new insights on the pathophysiological background, clinical presentation, and management of this condition. Aim of this review is also to provide advice for the clinical approach to avoid an undermanagement of T deficiency in MLWH and its possible short- and long-term consequences on quality of life, health status, and well-being.

## Clinical relevance of hypogonadism in MLWH

### Size of the problem

Low serum T is a common finding in MLWH even before the age of 50 (14). According to current literature data the prevalence of MLWH is highly variable, owing to the heterogeneity of patients’ age and different cut-off used to define low serum T, the method used for T measurement and the type of T considered for the diagnosis: total T (TT), direct and calculated free serum T (cFT) (the latter considering also serum SHBG changes), or both (cFT and TT) (15, 16, 18). Differences among studies are also due to methodological limitations such as the lack of standardization of the timing of blood samples in the morning for the assay of serum TT, the use of direct methods of FT measurement which are inaccurate (with the exception of equilibrium dialysis assay) (45) and the use of arbitrary cut-offs to define low serum T, and a small sample size of participants (16). Net of these limitations, the prevalence of overt hypogonadism in this population decreased over time, while the finding of subclinical hypogonadism is actually emerging.

Different studies have defined the prevalence of low serum T in MLWH in a range from 13 to 40% (16, 18). According to the results of a recent metanalysis, the prevalence of hypogonadism in MLWH subjects is of 26% (17). This prevalence is higher in studies that defined low serum T considering free serum T than in those that considered serum TT (17). The same results come from studies directly comparing the rate of prevalence of hypogonadism in MLWH obtained using cFT with TT (18, 28). These results are due to alterations of SHBG that are very common in MLWH (16, 28). SHBG, in fact, increases with advancing age at a higher rate compared to HIV-seronegative men (46) and accounts for reduced cFT when serum SHBG is elevated (16, 18, 28). From a pathophysiological standpoint, MLWH have higher circulating SHBG concentrations compared with those without HIV in cross-sectional studies (15), although the mechanisms for this remain unknown. To explain this difference, it has been hypothesized that systemic inflammation, that remains higher in MLWH even with effective antiretroviral therapy, may influence circulating SHBG concentrations (47). In addition, it is possible that systemic inflammation may affect glycosylation patterns of plasma proteins such as SHBG, which in turn may decrease the clearance of circulating SHBG in MLWH (47). Moreover, chronic HIV infection has been supposed to accelerate the aging process. Thus, aging and HIV may share similar underlying pathophysiological mechanisms that could potentially explain the higher levels of SHBG associated with both aging and HIV (47). The theoretical increase of SHBG has to be considered in the clinical practice during the diagnostic-therapeutic work-up of hypogonadism in MLWH (see paragraph “Diagnosis”).

### Clinical impact of hypogonadism in MLWH

Considering the high prevalence of hypogonadism in MLWH and the early onset of the disease (14, 17, 31), T deficiency is expected to significantly impact on several aspects of MLWH general health and well-being including bone health, sexual life, quality of life and physical performance/vigor. The putative impact of hypogonadism on all these aspects is of particular concern since most of them are common in MLWH independently from T deficiency and are due to other factors. Accordingly, sexual dysfunction (48–50), osteoporosis (27, 51), body composition changes (52), metabolic complications (i.e. impaired glycemic and lipid profile) and lower overall quality of life (53, 54) are common in MLWH.

## Classifications of hypogonadism in MLWH

The most common clinical condition of hypogonadism diagnosed in MLWH is secondary hypogonadism characterized by low than normal or inappropriately normal serum luteinizing hormone (LH) in presence of serum T (TT or cFT) below the normal range (i.e. hypogonadotropic hypogonadism) (14, 15, 18, 55). Primary (hypergonadotropic) hypogonadism due to testicular dysfunction is less common and is characterized by serum LH higher than normal together with serum T (TT or cFT) below the normal range (14, 15, 18, 55). Among MLWH with overt hypogonadism, secondary hypogonadism ranges from 80% (14, 18) to 100% (55) of cases, while primary hypogonadism represent 14% to 37% of cases or less, according to different studies (14, 15, 18, 55).

Compensated, or subclinical, hypogonadism (56) represents a clinical entity characterized by normal serum T (TT or cFT), but increased gonadotropins. High LH may be a biomarker for readjustment of the set point of hypothalamic-pituitary-gonadal axis feedback in MLWH, similarly to aging, to compensate for deficiencies in testicular function and/or defective T feedback at the central level (56). Compensated hypogonadism is very common (about 50%) among MLWH (14, 18). Due to the possibility of an eventual progression from subclinical into overt hypogonadism (56), this state warrants continued monitoring to prevent or preempt further deterioration.

## Determinants of hypogonadism

The pathogenesis of hypogonadism in MLWH remains to be fully elucidated. Different hypotheses on the underlying pathophysiological mechanisms have been provided, but their cause-effect relationships need to be substantiated by further evidence. With the exception of few well-identified causes, the etiology of hypogonadism in MLWH seems to be multifactorial where a multitude of factors concur in different manners to T reduction. This multifactorial view related to T deficiency has been recently named as functional hypogonadism (i.e. secondary hypogonadism) (42, 43, 57).

Among the classical factors correlated with hypogonadism in men, most, such as age, ethnicity, body mass index, visceral adiposity indexes, alcohol consumption, cigarette smoking, and sedentary lifestyle, seem to have a minor role in reducing serum T levels (**Table 1**) (14, 31, 58–60). Concerning age, hypogonadism is common in young and middle aged MLWH (14, 17, 18) while in HIV seronegative men occurs later in life (61) and is rare before 40 (62).

**Table 1:** Main factors involved in the pathogenesis of hypogonadism in MLWH subdivided into classical and HIV-specific factors.

Classical factors	HIV -related factors
Age	HIV-infection *per se*
Ethnicity	HIV-infection duration
Body mass index	HAART duration
Waist circumference	Frailty and multimorbidity
Visceral adiposity	Premature aging
Physical activity	HBV and/or HCV co-infection
Alcohol consumption	Testicular opportunistic infections
Cigarette smoking	Abuse of opiates
Hypertension	Changes of body composition (lipodystrophy)
Diabetes and pre-diabetes	

MLWH, men living with HIV; HAART, highly active antiretroviral therapy; HBV, hepatitis B virus; HCV, hepatitis C virus.

In parallel with traditional risk factors for hypogonadism, several HIV-related peculiar factors have been proven to be involved in the pathogenesis of T deficiency in MLWH. Actually, the association between serum T levels and HAART drugs or HIV-related parameters (e.g. viral load, CD4 count, duration of HIV) has been reported but it lacks of compelling evidence (14, 58, 63) (**Table 1**).

It is important to specify that the lack of strong association between T levels and different risk factors, especially from the classical category, does not mean that these factors are not involved in the development of hypogonadism. In HIV setting the co-existence of many other peculiar factors can mask, from a statistical point of view, the weight of traditional factors in T deficiency pathogenesis (14, 31).

A small number of MLWH with hypogonadism have an evident known cause of T deficiency. Theoretically, any organic recognized cause of T deficiency such as hypothalamic-pituitary diseases (pituitary adenomas, other tumors, inflammatory diseases, etc.) and/or surgery or irradiation, rare congenital diseases and primary testicular disorders may present also in MLWH, thus leading to a diagnosis of organic or classical hypogonadism (57). Similarly, the use of opiates is a well-known cause of hypogonadism since opioids inhibit gonadotropins production by acting both at hypothalamic and pituitary level (64, 65) and data are available showing higher rate of hypogonadism in MLWH using opiates compared to non-users (66–68). Use of injection drugs, and hepatitis C and/or B co-infection seem to be only slightly associated with lower serum T levels in several studies (**Table 1**) (59, 60, 63, 66). Furthermore, in MLWH with uncontrolled HIV infection, rare opportunistic infections of the testis may lead to primary (hypergonadotropic) hypogonadism due to testicular damage (69).

Outside the context of the abovementioned organic diseases leading to hypogonadism, low serum T may be related to advancing age and to the general health status in terms of comorbidities accumulation and chronic diseases (**Table 1**) (31, 33). As for the male general population, the emergent condition of functional hypogonadism, in contrast to the organic or classical hypogonadism, is gaining credence as a defined clinical entity and diagnosis even in this HIV setting. Functional hypogonadism is defined as the co-existence of low serum T concentrations occurring in the absence of both intrinsic structural hypothalamic-pituitary-gonadal axis disease and of specific pathologic conditions suppressing axis’ functionality (43). Hence, in MLWH functional forms of hypogonadism without a clear-cut cause, that result in central (or possibly mixed) forms, are the most frequent. Evidence suggests that a poor health status and multimorbidity are directly related to low serum T (TT and cFT) (31, 33). It is well-known that MLWH develop several comorbidities due to both HIV infection and undesired effects of HAART (1) that contribute to a sort of accentuated aging in young to middle aged MLWH (4, 5) together with poor health and HIV-related frailty (70). Conditions like impaired glucose metabolism (diabetes or pre-diabetes), dyslipidemia, hypertension, and surrogates of cardiovascular diseases (e.g. coronary artery calcification and carotid intima-media thickness) all seem to concur to testosterone deficiency in MLWH, despite not being strong predictors (31, 60, 63, 71). Frailty represents a state of vulnerability to adverse health outcomes occurring in response to a wide variety of stressors. Although frailty was originally described among community-living populations over 65 years old, frailty has been studied over the past decades in other populations including MLWH. Recent guidelines recommend that all MLWH over 50 be screened annually for frailty (72), although there is lack of consensus regarding which frailty measure, among many available, is reliable and simple enough to use in routine clinical settings (73). Actually, the Frailty Index (FI) is one of the most used strategies to assess frailty in HIV; it is useful to predict future risk of adverse outcome and it represents the proportion of health deficits present out of a group of condition (74). Given the growing body of literature stating that frailty may be transitional, the recognition and management of related risk factors will help to mitigate the development of frailty.

For all these reasons recent guidelines on male hypogonadism draw attention to HIV infection as an important factor involved in T deficiency and to MLWH as a population who needs to be considered for hypogonadism even at a young age and deserving specific work up if hypogonadal symptoms are present (25, 26).

## Clinical management

### Clinical presentation

**Figure 1:**
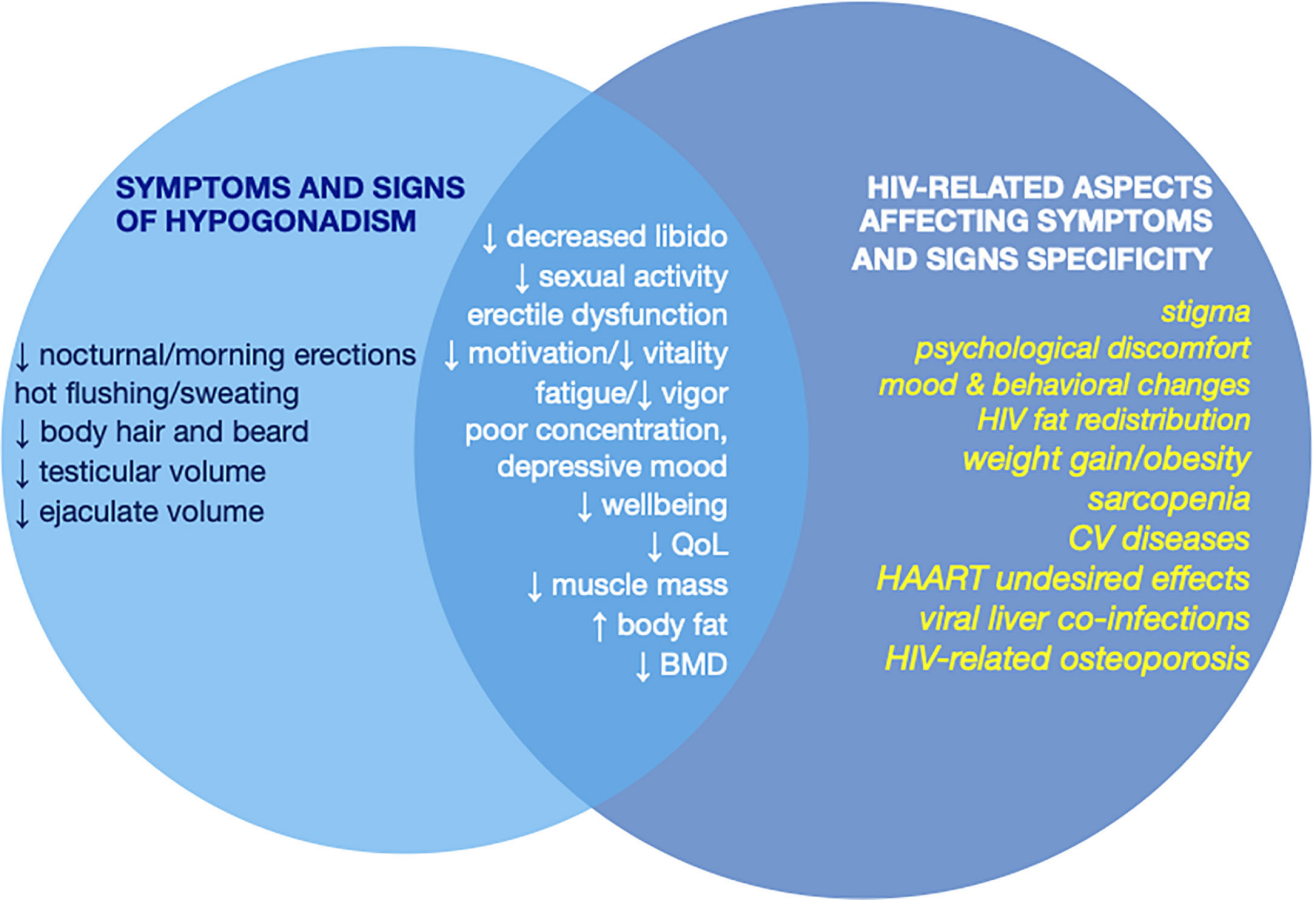
Symptoms and signs of hypogonadism and their specificity in MLWH according to the overlap with clinical manifestations of HIV, HAART, and HIV-related comorbidities that may affect specificity of the clinical presentation of hypogonadism. MLWH, men living with HIV; QoL, quality of life; BMD, bone mineral density; CV, cardiovascular; HAART, highly active antiretroviral therapy.

Due to the high prevalence of hypogonadism in MLWH, possible signs of hypogonadism should be carefully investigated in these patients to rule in/out a diagnosis of T deficiency (**Figure 1**). As for the general population, indeed, the diagnosis of hypogonadism is recommended only on the basis of the presence of clinical symptoms or signs of T deficiency in combination with consistently low morning serum T concentrations (25, 26, 43).

However, as for other systemic diseases (38) specificity of most of the symptoms and signs of hypogonadism is poor due to the overlap of clinical features with those of HIV infection, HAART undesired effects, and HIV-related comorbidities (16) (**Figure 1**). Among signs and symptoms of hypogonadism that are less specific in MLWH those related to mood, behavior, psychological issues and physical performance need to be mentioned. Similarly, the prevalence of osteopenia and osteoporosis is very high in MLWH (51, 75, 76) and bone mass reduction occurs often outside the context of hypogonadism even in presence of normal levels of circulating T (14, 77) (**Figure 1**). The same happens for erectile dysfunction whose prevalence is very high in MLWH but does not differ between hypogonadal MLWH and MLWH with normal serum T (14, 49, 50). Similarly, body composition changes in MLWH resemble those of hypogonadal men and are characterized by weight gain, increased fat mass (especially visceral fat) and reduced fat free mass and muscle mass (78) and are worsened by concomitant low serum T. In contrast, other elements such as reduced sleep-related/morning erection, hot flushes/sweating, reduced testis volume, and decrease of body hair and beard are more specific for hypogonadism and less influenced by other factors (79–83) (**Figure 1**).

### Diagnosis

**Figure 2:**
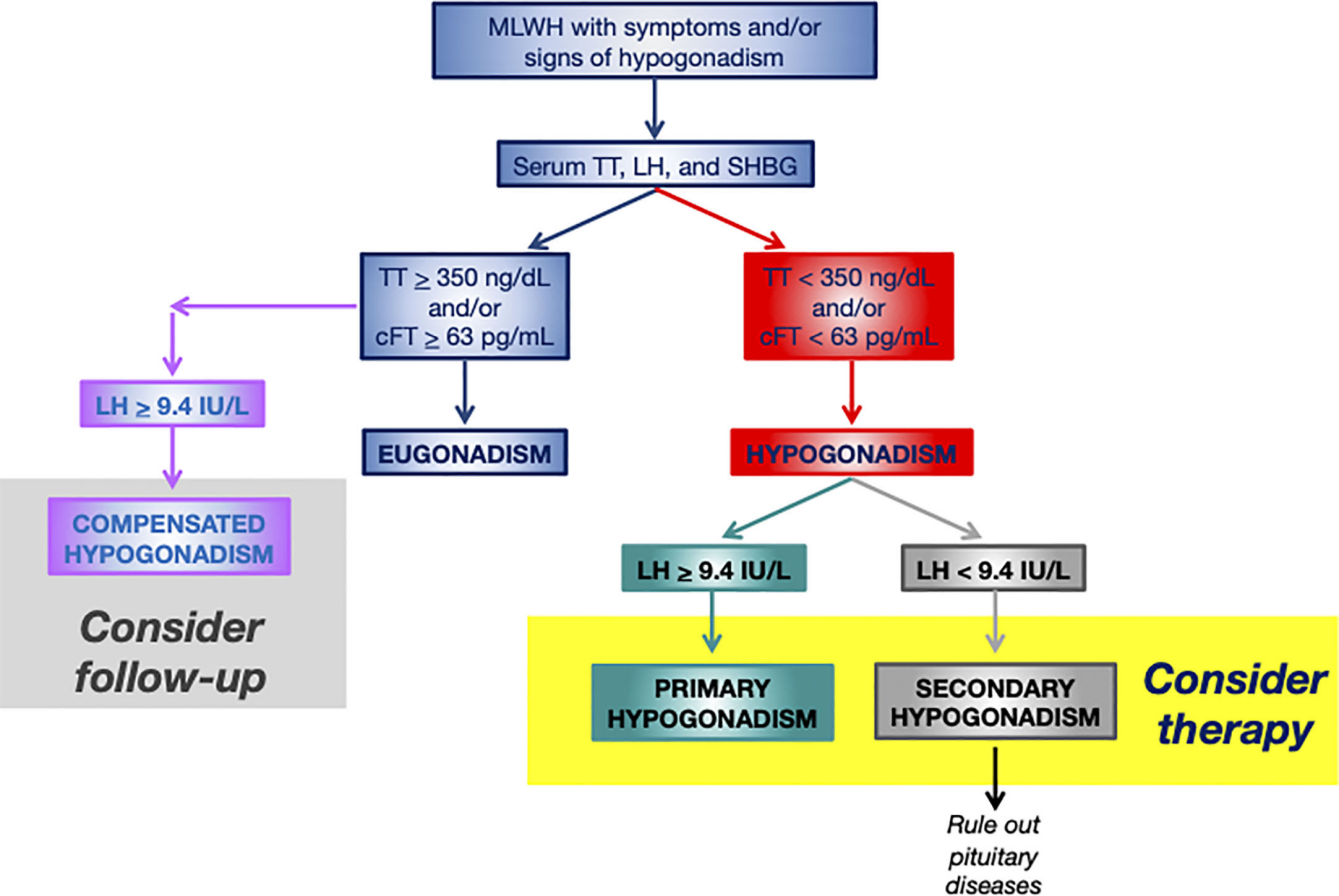
Flowchart for the diagnosis and management of hypogonadism in MLWH. More restrictive thresholds of 300 ng/dL for TT and of 50 pg/mL for cFT should be considered for older MLWH (>60 years). MLWH, men living with HIV; TT, serum total testosterone; LH, luteinizing hormone; SHBG, sex hormone binding globulin; cFT, calculated free testosterone.

T concentrations must be checked in all MLWH with clinical manifestations of T deficiency by performing biochemical tests (16, 26) (**Figure 2**). Notwithstanding the high prevalence of hypogonadism, there is no evidence at present suggesting screening for hypogonadism all MLWH indiscriminately (16), but at the same time clinical examinations should not be delayed in presence of at least one symptom of T deficiency since hypogonadism is common even in young to middle aged MLWH (14, 31). Again, patients with documented clinical conditions that are associated to hypogonadism should be screened. Biochemical testing, in fact, is mandatory in MLWH with osteopenia or osteoporosis, sexual dysfunctions (particularly erectile dysfunction) according to guidelines (25, 26, 43, 84).

Measurement of serum LH, TT, SHBG for the calculation of FT (cFT) are first line biochemical tests (**Table 2**, **Figure 2**). These measurements allow obtaining information on serum TT and cFT as well as to establish the origin (primary *vs.* secondary) of hypogonadism in case of low serum circulating T (14, 25, 26, 56) (**Figure 2**). SHBG is mandatory in MLWH since the lack of information on cFT may lead underestimating hypogonadism (18, 25, 28, 55, 85). The importance of measuring serum SHBG in MLWH was already highlighted by the Endocrine Society guidelines on male hypogonadism (25) which focused on subgroups of men who are at higher risk of SHBG abnormalities including MLWH. Recently, serum SHBG measurement has been suggested for all men with suspected hypogonadism by the guidelines of the Italian Society of Andrology and Sexual Medicine even outside the context of specific diseases (e.g. HIV, liver disease) in order to avoid the underestimation of the diagnosis of hypogonadism in men complaining symptoms of testosterone deficiency (26). Serum T threshold below which biochemical hypogonadism is diagnosed is not universally established. Various thresholds have been proposed overtime ranging from 300 to 350 ng/dL (10 to 12 nmol/L) for serum TT and from 50 to 63 pg/mL (170 to 220 pmol/L) for serum cFT according to different guidelines recommendations (25, 26). Since hypogonadism occurs in MLWH at a young age (often before 50 years) (14, 17, 18) a higher threshold of 350 ng/dL (12 nmol/L) for TT and of 63 pg/mL (220 pmol/L) seem to be more appropriate and in line with higher physiological serum levels in this range of age (**Figure 2**). Starting from serum TT and SHBG, cFT is easy to be calculated by using the Vermeulen formula (86) which is the most accurate among methods for cFT calculation (87) (http://www.issam.ch/freetesto.htm).

**Table 2:** First and second line clinical examinations useful for the work-up of hypogonadism in MLWH.

First line examinations	Second line examinations
**Serum Parameters:** TT LH SHBG cFT*	**Secondary Hypogonadism:** Serum Prolactin Other tests for pituitary function Pituitary MRI **Primary Hypogonadism:** Testes ultrasound **Morbidities related to hypogonadism:** IIEF-15 DXA Bone metabolism function (calcium phosphorous, PTH, vitamin D, markers of bone turnover) X-ray of the spine according to clinical symptoms (e.g., back pain) and/or bone mineral density status **Suitability before starting T therapy** Serum PSA Hematocrit

*for calculating cFT use this link: http://www.issam.ch/freetesto.htm.

MLWH, men living with HIV; TT, serum total testosterone; LH, luteinizing hormone; SHBG, sex hormone-binding globulin; cFT, calculated free testosterone; MRI, magnetic resonance imaging; IIEF, International Index of Erectile Function; DXA, dual X-ray absorptiometry; PSA, prostate specific antigen.

Some methodological issues are important for the diagnosis of hypogonadism: 1) serum T should be measured on serum blood samples taken in the morning between 7 and 10 am; 2) clinician should be aware of the accuracy and limits of the method used for serum T measurement; 3) T deficiency should be verified at least twice before confirming the diagnosis of hypogonadism; and 4) direct methods for the measurement of FT with commercially available assays should be avoided due to its high inaccuracy, with the exception of equilibrium dialysis (16, 45).

Second-line evaluations include pituitary function tests (especially serum prolactin) in case of confirmed secondary (hypogonadotropic) hypogonadism and magnetic resonance imaging of the pituitary if a hypothalamic-pituitary disease is suspected on the basis of severe T deficiency or concomitant other clinical features of pituitary diseases (25, 26). Furthermore, a specific bone health assessment should be performed in case of diagnosis of hypogonadism according to available guidelines (84). In case of primary hypogonadism ultrasound of the testes is strongly suggested (**Table 2**).

A detailed interview on patient’s current or previous use of drugs and/or substance abuse (e.g. androgens, other anabolic steroids, gonadotropins) is mandatory in order to exclude iatrigenic pharmacological causes of T deficiency whose removal may revert hypogonadism.

Finally, a comprehensive evaluation of MLWH with documented hypogonadism should include specific clinical examinations useful to investigate sexual function, bone health, and to check contraindications to T therapy (**Table 2**).

## Therapy of hypogonadism in MLWH

Testosterone replacement therapy (TRT) should be considered in MLWH with documented low serum T (TT or cFT) and concomitant symptoms and signs of hypogonadism (**Figure 2**). On the contrary, T therapy should not be administered in the attempt to treat HIV-related conditions such as osteopenia/osteoporosis, sarcopenia, frailty, obesity, diabetes mellitus type 2, and sexual dysfunction to improve disease outcomes if serum T is in the normal range since its efficacy is not proved (26).

TRT as monotherapy is not enough for MLWH with a confirmed diagnosis of hypogonadism and concomitant erectile dysfunction persisting during TRT, osteopenia/osteoporosis, diabetes mellitus, and other comorbidities; in this clinical context specific pharmacological treatments (e.g. PDE5 inhibitors, bone active drugs, antidiabetic agents) should be added to TRT (26, 84).

Reversibility of secondary hypogonadism should be taken into account in some particular circumstances (e.g. obesity, acute diseases) and TRT may be postponed or discontinued to check recover of pituitary gonadal axis function after having treated and/or reversed the underlying disease/condition (e.g. after significant weight loss due to lifestyle changes) (38). TRT remains a good treatment in case of failure of primary intervention (lifestyle changes or pharmacological).

All pharmacological preparations and formulations of T available on the market are effective and their choice depends on several factors including overall patient’s preference (16, 25, 26) (**Table 3**). The dose should be titrated and established according to several issues such as age, severity of hypogonadism, health status of the patient as for HIV-uninfected subjects (16, 25, 26) (**Table 3**).

**Table 3:** Exogenous T preparations and formulations and factors involved in the choice of T dosage and formulation.

Preparations	Factors to be considered for choosing T formulations and dosages
Oral formulations: *T undecanoate* Intramuscular injections: *T enanthate, T cypionate, T undecanoate* Skin disposal: *Transdermal T patches, T gel*	Age Serum T at baseline/severity of hypogonadism Health individual conditions Potential reversibility of hypogonadism Cause of hypogonadism Patient’s CV risk

T, testosterone; CV, cardiovascular.

Patient’s health status is of relevance especially in MLWH since they display accelerated aging, and frailty (1, 2, 5, 31, 33) and TRT resulted associated to increased mortality in frail older men (88). Since MLWH are at higher risk of cardiovascular diseases and major adverse cardiovascular events (MACE) (89) and risk/benefits of TRT should be addressed before starting T therapy in MLWH through an accurate assessment of global cardiovascular risk (16, 26) bearing in mind that TRT is potentially harmful in men with recent MACE, documented severe heart failure, and history of thromboembolism (26).

It is still a matter of debate if TRT should be administered to all MLWH with low serum T since i) hypogonadism may be reversible as in case of weight loss in obese/overweight patients; ii) serum T may be an epiphenomenon due to poor health status and frailty and may be an adaptive condition aiming to sparing energy; iii) TRT maybe harmful in some patients (16, 31, 33, 38). Recently, the results from the PUSH study! have suggested that there are no additional side effects when TRT is administered to MLWH (90), but evidence is still lacking since no studies have been addressed to TRT safety in MLWH compared to HIV-uninfected men (16, 91). As for male general population, TRT is contraindicated in presence of active prostate and breast cancer as well as in men searching for fatherhood (25, 26).

## Follow-up

Hypogonadal MLWH treated with TRT needs to be monitored overtime mainly by measuring serum TT and cFT. Follow-up should include the evaluation of TRT efficacy on hypogonadal symptoms (e.g. improvement of sexual function, quality of life, body weight and body composition), and TRT safety by checking periodically (every six months at the begin of therapy and then yearly) prostate specific antigen (PSA) and hematocrit (26). In addition, therapy outcomes that are expected to be modified by TRT should be periodically monitored. Sexual function and activities should be carefully monitored to record improvements and useful tools, such as IIEF or other questionnaires, are available to objectify changes (50). TRT is able to prevent bone mass loss and TRT increase BMD even though no data are available on TRT efficacy in preventing osteoporotic fractures (84, 92). In MLWH with hypogonadism TRT improves body composition as demonstrated in a metanalysis including several studies (17) thus confirming data coming from HIV-uninfected men (26, 93, 94). Changes in body weight and waist circumference should be monitored in the follow-up.

## Conclusions

T deficiency is common among MLWH. Since its clinical presentation could dispense different pitfalls due to the non-specificity of signs and symptoms that may overlap with other conditions frequent in MLWH, the real prevalence of male hypogonadism in HIV tends to be underestimated. Even research studies have provided quite heterogeneous data mainly suffering of methodological limitations, and recently stating a mean prevalence of hypogonadism of 26% in MLWH.

Following the advances reached in HIV medicine over the last decades, changes in the pathophysiological mechanisms involved in the onset of male hypogonadism have been suggested. There is emerging evidence that advocates a functional nature of hypogonadism in MLWH, where HIV-related and non-related determinants such as sex steroids, general health status, and chronic systemic comorbidities, concur all together in determining the serum T decrease.

Whereas clinical interview to assess the presence of signs suggestive of hypogonadism should be routinely part of the clinical work-up of MLWH, the evaluation of the hypothalamic-pituitary-gonadal axis by biochemical analysis threshold be reserved to MLWH for whom low T levels are suspected. In these patients, measurements of gonadotropins, serum TT and SHBG are necessary for an accurate assessment of gonadal function, and a correct identification of not only overt, but even compensated forms of hypogonadism. Assessing sexual health in MLWH together with other signs and symptoms of hypogonadism is crucial to select patients for biochemical evaluation and to avoid underestimation of hypogonadism.

Finally, a multidisciplinary management is suggested involving both the specialist of infectious diseases and the endocrinologist (or andrologist) who could better tailor the choice of T treatment, when necessary. Tailoring the risks benefits ratio to each MLWH patient with documented hypogonadism remains challenging as the decision to start TRT as well.

## Author contributions

SV and VR contributed to conception and literature search, wrote the first draft of the manuscript, and provided revisions. Both authors read and approved the submitted version. All authors contributed to the article.
